# Stem Cell Therapy for Diabetic Erectile Dysfunction in Rats: A Meta-Analysis

**DOI:** 10.1371/journal.pone.0154341

**Published:** 2016-04-25

**Authors:** Mingchao Li, Hao Li, Yajun Ruan, Tao Wang, Jihong Liu

**Affiliations:** Department of Urology, Tongji Hospital, Tongji Medical College, Huazhong University of Science and Technology, Wuhan, Hubei, China; Max-Delbrück Center for Molecular Medicine (MDC), GERMANY

## Abstract

**Introduction:**

Stem cell therapy is a novel method for the treatment of diabetic erectile dysfunction (ED). Many relative animal studies have been done to evaluate the efficacy of this therapy in rats.

**Aims:**

This meta-analysis was performed to compare the efficacy of different stem cell therapies, to evaluate the influential factors and to determine the optimal stem cell therapeutic strategy for diabetic ED.

**Methods:**

We searched the studies analyzing the efficacy of stem cell therapy for diabetic ED in rats published before September 30, 2015 in PubMed, Web of Science and EBSCO. A random effects meta-analysis was conducted to assess the outcomes of stem cell therapy. Subgroup analysis was also performed by separating these studies based on their different characteristics. Changes in the ratio of intracavernous pressure (ICP) to mean arterial pressure (MAP) and in the structure of the cavernous body were compared.

**Results:**

10 studies with 302 rats were enrolled in this meta-analysis. Pooled analysis of these studies showed a beneficial effect of stem cell therapy in improving erectile function of diabetic rats (SMD 4.03, 95% CI = 3.22 to 4.84, *P*< 0.001). In the stem cell therapy group, both the smooth muscle and endothelium content were much more than those in control group. There was also significant increase in the expression of endothelial nitric oxide synthase (eNOS) and neuronal nitric oxide synthase (nNOS), the ratio of smooth muscle to collagen, as well as the secretion of vascular endothelial growth factor (VEGF). Besides, apoptotic cells were reduced by stem cell treatment. The subgroup analysis indicated that modified stem cells were more effective than those without modification.

**Conclusions:**

Our results confirmed that stem cell therapy could apparently improve the erectile function of diabetic rats. Some specific modification, especially the gene modification with growth factors, could improve the efficacy of stem cell therapy. Stem cell therapy has potential to be an effective therapeutic strategy for diabetic ED.

## Introduction

Diabetic erectile dysfunction (ED) is a major health problem which has a strong impact on the quality of life of patients and the harmony of their families. It was reported that the prevalence of ED in diabetic patients was about three times greater than that in nondiabetic men [1]. Additionally, ED in patients with diabetes mellitus is more severe and refractory compared with that in nondiabetic patients [2]. The dysfunction of endothelium, decrease of smooth muscle content, neural degeneration and fibrosis produced by diabetes contribute to the formation of erectile dysfunction [3]. Although phosphodiesterase type 5 inhibitors (PDE5i) are effective for the treatment of ED in most patients, its efficacy is much lower for diabetic ED [4]. This is probably caused by the decrease in nitric oxide (NO) production resulting from endothelial dysfunction [5]. In this condition, some alternative therapeutic strategies are being explored to treat diabetic ED more effectively, such as gene therapy and stem cell therapy.

Stem cell therapy is a promising treatment for diabetic ED. Several kinds of stem cells, such as bone marrow mesenchymal stem cells (BMSC), adipose tissue-derived stem cells (ADSC) and urine-derived stem cells (USC), have been used to treat ED in animal models [6–8]. Stem cells are able to differentiate into various cell types, including vascular endothelial cells, smooth muscle cells (SMC) and neurons. Moreover, they can secrete paracrine factors which may enhance cell survival and angiogenesis [9]. Intracavernous transplantation of stem cells has been reported to have beneficial effects on erectile function of diabetic rats in many studies. The endothelial function and the content of smooth muscle cells or pericytes in the cavernous body were reported to increase in some of these studies. In order to enhance the efficacy of stem cell therapy, stem cells modified with growth factor genes were utilized in several studies, which showed that the improvement of erectile function in groups treated with modified stem cells was better than that treated with unmodified ones [6–8, 10–16].

Although studies suggested that stem cell therapy might be beneficial for diabetic ED, the optimal strategy has not been determined up to now. Confirming the most effective strategy is meaningful for the future experimental design, which will also provide some new ideas for the treatment of diabetic erectile dysfunction in clinical practice. The present meta-analysis was conducted to compare the efficacy of different stem cell therapies in improving diabetic erectile dysfunction in rats and determine the optimal strategy.

## Methods

### Search methods

We searched the pre-clinical studies analyzing the efficacy of stem cell therapy for diabetic ED published before September 30, 2015 in PubMed, Web of Science and EBSCO. The following search strategy was used: (Erectile dysfunction) and (stem cell) and (diabetes) and (rat). Secondary references were also reviewed. Only these papers in English were included. Papers from different databases were examined carefully to exclude duplications.

### Selection criteria

Studies satisfying the following criteria can be included in our meta-analysis: (1) randomized controlled trial; (2) the objects of the study were diabetic rats; (3) stem cells were used exclusively to improve the erectile function; and (4) erectile function is evaluated by ICP/MAP via electric stimulation of the cavernous nerve. We made these including criteria by using the criteria from Haitao Shan, et al. [17] as reference.

### Data extraction

Data of the included studies were extracted from all available sources, including tables and graphs. When data were presented only graphically and without responding from the authors, values were obtained via quantitative methods by using the software of GetData Graph Digitizer (S. Fedorov). Two authors of our study extracted these data separately. Discrepancies between the two authors were resolved by consensus.

### Quality Assessment

The methodological quality of the included studies was evaluated by two authors. Disagreements were resolved by consensus. The assessment of quality was conducted with the following 9 criteria: (1) publication in a peer-reviewed journal; (2) randomization of the experiment; (3) blinded outcome assessment; (4) compliance with animal welfare regulations; (5) identification of the stem cell phenotype; (6) stem cells were labeled with specific markers; (7) evaluation of erectile function with apomorphine (APO) test before the injection of stem cells; (8) sample size calculation; and (9) detection of the structural changes in the cavernous body. Each criterion was given one point. The studies were classified into 3 quality categories according to the points (high quality: 7–9 points; moderate quality: 4–6 points; and low quality: 0–3 points).

### Statistical analysis

We used Review Manager 5.3 (The Nordic Cochrane Center) to analyze the outcome data. The primary outcome was presented in the form of the standardized mean difference (SMD) with 95% confidence intervals (CI), which represented the differences in erectile function and structural changes of the cavernous body between the stem-cell treatment groups and control groups. Each trial consisted of 1 control group and ≥1 treatment group. When there was more than one treatment group in one study, the number of rats in the control group was divided equally by the number of treatment groups. In case of multiple measurements in different time, the data of the last measurement were used for analysis.

Subgroup analysis was also performed to examine the influence of several factors on the efficacy, such as the type of stem cells (BMSCs, USCs, or ADSCs), number of injected cells (<1×10^6^ or ≥1×10^6^), time point of cell-injection after the diabetic model establishment (within 8 weeks or beyond 8 weeks), follow-up time after stem cell injection (<4 weeks or ≥4 weeks), stem cell sources (autologous, allogeneic or xenogeneic), diabetes types (type 1 or type 2), and modification of stem cells (with or without modification).

We used a random effects model to avoid heterogeneity. The results of the meta-analysis were presented as forest plots, in which the studies were arranged according to the year of study. Besides, a funnel plot was utilized to examine a potential publication bias.

## Results

### Search results and characteristics of included studies

**Fig 1:**
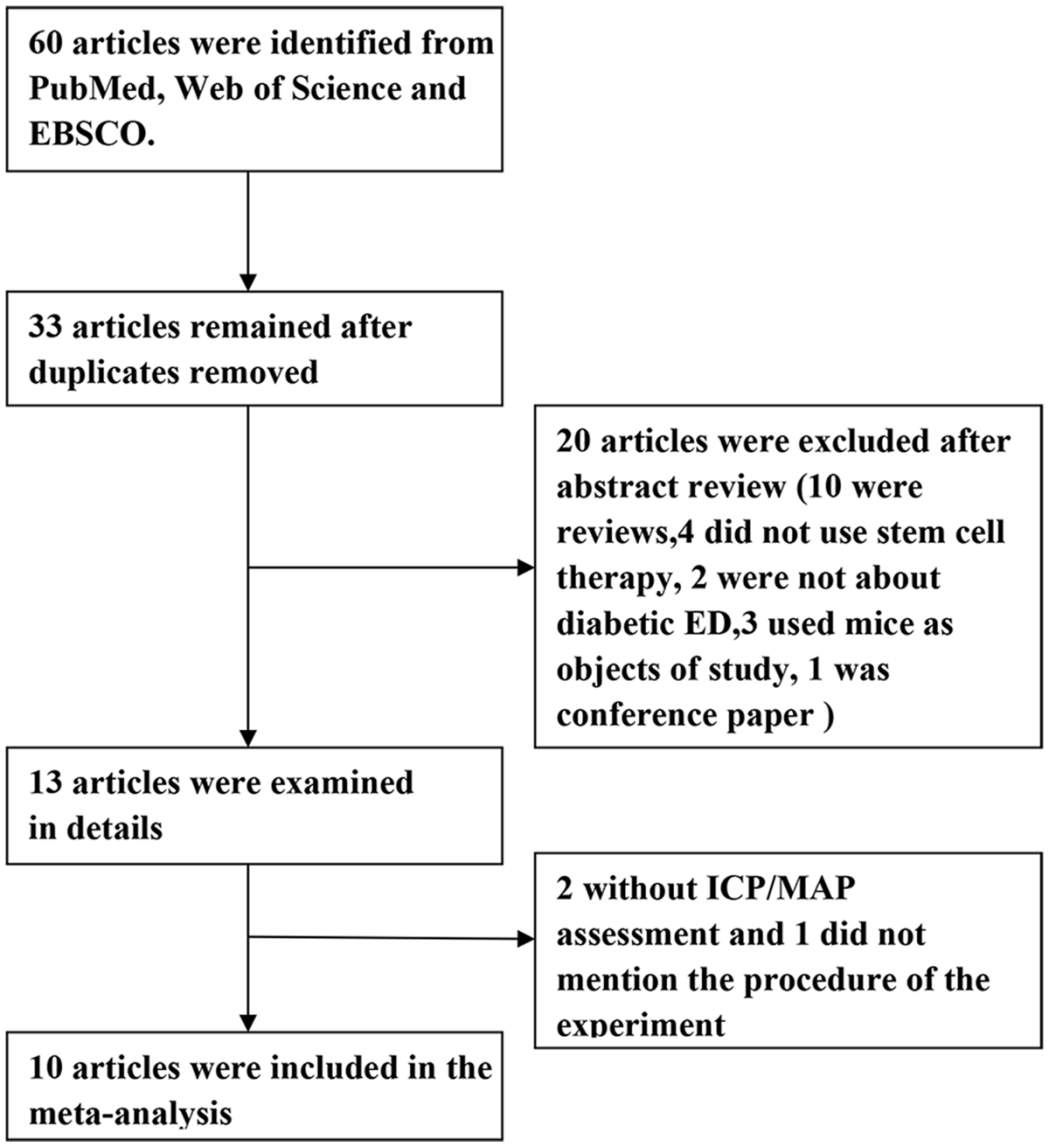
Flowchart of study selection.

Electronic searching identified a total of 60 publications, of which 10 articles met our inclusion criteria and were enrolled in the meta-analysis (Fig 1). And 302 rats in these studies were included. Characteristics of the enrolled studies are described in Table 1.

**Table 1:** Characteristics of Included Studies.

Year	First author	Rat species	Age of rat(weeks)	Sample size(n)	Cell type	Cell number	Modification	Injection time(weeks)*	Follow-up time(weeks)
**2010**	**MM Garcia**	**ZDF**	**10**	**20**	**Autologous ADSC**	**1×10**^**6**^	**No**	**23**	**3**
**2011**	**Xuefeng Qiu**	**SD**	**10**	**27**	**Allogeneic BMSC**	**4×10**^**5**^	**No**	**8**	**4**
**2012**	**C. Sun**	**SD**	**10**	**56**	**Allogeneic BMSC**	**5×10**^**5**^	**No**	**8**	**2**
**2012**	**Hiroaki Nishimatsu**	**Wistar**	**6**	**32**	**Allogeneic ADSC**	**5×10**^**5**^	**EGM**	**6**	**4**
**2012**	**Xuefeng Qiu**	**SD**	**10**	**46**	**Allogeneic BMSC**	**5×10**^**5**^	**VEGF**	**8**	**4**
**2013**	**Guihua Liu**	**SD**	**10**	**30**	**Allogeneic ADSC**	**1×10**^**6**^	**VEGF**	**8**	**4**
**2014**	**Bin Ouyang**	**SD**	**No mention**	**65**	**Human USC**	**1×10**^**6**^	**FGF2**	**8**	**4**
**2014**	**Y. He**	**SD**	**6 to7**	**43**	**Allogeneic BMSC**	**1×10**^**6**^	**KCNMA1**	**8**	**2**
**2015**	**Tao Liu**	**SD**	**No mention**	**40**	**Autologous ADSC**	**2×10**^**6**^	**HGF**	**13**	**4**
**2015**	**Xiyou Wang**	**SD**	**10**	**62**	**Allogeneic ADSC**	**1×10**^**6**^	**Hypoxia**	**8**	**1**

ZDF: Zucker Diabetic Fatty; SD: Sprague-Dawley; ADSC: adipose-derived stem cells; BMSC: bone marrow mesenchymal stem cells; USC: urine-derived stem cells; VEGF: vascular endothelial growth factor; FGF2: fibroblast growth factor 2; HGF: hepatocyte growth factor; EGM: endothelial growth medium; KCNMA1: calcium-activated potassium channel subunit alpha-1.

*The cell-injection in ZDF rats was at the age of 23w, while in other studies the cell-injections were at 6 weeks, 8 weeks or 13 weeks after STZ injection.

Nine of the studies used streptozocin (STZ) injection to induce diabetes in rats and the other one used the Zucker Diabetic Fatty (ZDF) rats, which had genetic diabetes. The diabetic rats enrolled were confirmed by blood glucose measurement. Stem cells were injected into the cavernous body 8 weeks after STZ injection in seven studies, 6 weeks in one study and 3 months in one study. In the study with ZDF rats, stem cells were injected at the age of 23 weeks. Six studies injected 1×10^6^ cells or more into the penis, while four studies chose 5×10^5^ cells or less as injection dosage. Altogether, three kinds of stem cells (ADSCs, BMSCs and USCs) were used in these studies with or without modification. The modification methods include genetical modification with growth factors (VEGF; fibroblast growth factor 2, FGF2; and hepatocyte growth factor, HGF) or ion channel protein (calcium-activated potassium channel subunit alpha-1, KCNMA1), cell culture in endothelial growth medium (EGM) and hypoxia precondition. The follow-up time after injection varied from 1 week to 30 days in different studies.

In all the studies, erectile function was evaluated via the electric stimulation on the cavernous nerve after anesthesia and the results were presented in the form of ICP/MAP. Besides the erectile function, the histological changes and molecular changes were also measured in nine of the studies, including the content of endothelial cells and smooth muscle cells, eNOS, nNOS, the ratio of intracavernous smooth muscle to collagen, apoptosis and so on.

### Quality of the included studies

According to results of the quality assessment, the quality of the included studies was of a relatively high level. Three of the studies were of high quality and seven of them were of moderate quality (Table 2).

**Table 2:** Quality assessment of studies.

Year	First author	Random assignment	Blind assessment	Sample size calculation	APO test	Animal welfare	Phenotype identification	Structural changes	Cell label	Peer evaluation	Total
**2010**	**MM Garcia**	**1**	**1**	**0**	**0**	**1**	**0**	**1**	**1**	**1**	**6**
**2011**	**Xuefeng Qiu**	**0**	**0**	**0**	**0**	**1**	**1**	**1**	**1**	**1**	**5**
**2012**	**C. Sun**	**1**	**0**	**0**	**0**	**1**	**1**	**1**	**1**	**1**	**6**
**2012**	**Hiroaki Nishimatsu**	**0**	**0**	**0**	**0**	**1**	**1**	**1**	**1**	**1**	**5**
**2012**	**Xuefeng Qiu**	**1**	**0**	**0**	**0**	**1**	**1**	**1**	**1**	**1**	**6**
**2013**	**Guihua Liu**	**0**	**1**	**0**	**1**	**1**	**1**	**1**	**1**	**1**	**7**
**2014**	**Bin Ouyang**	**1**	**1**	**0**	**1**	**1**	**1**	**1**	**1**	**1**	**8**
**2014**	**Y. He**	**1**	**0**	**0**	**1**	**0**	**1**	**0**	**1**	**1**	**5**
**2015**	**Tao Liu**	**0**	**0**	**0**	**0**	**1**	**1**	**1**	**1**	**1**	**5**
**2015**	**Xiyou Wang**	**1**	**0**	**0**	**1**	**1**	**1**	**1**	**1**	**1**	**7**

APO: apomorphine.

### Meta-analyses

**Fig 2:**
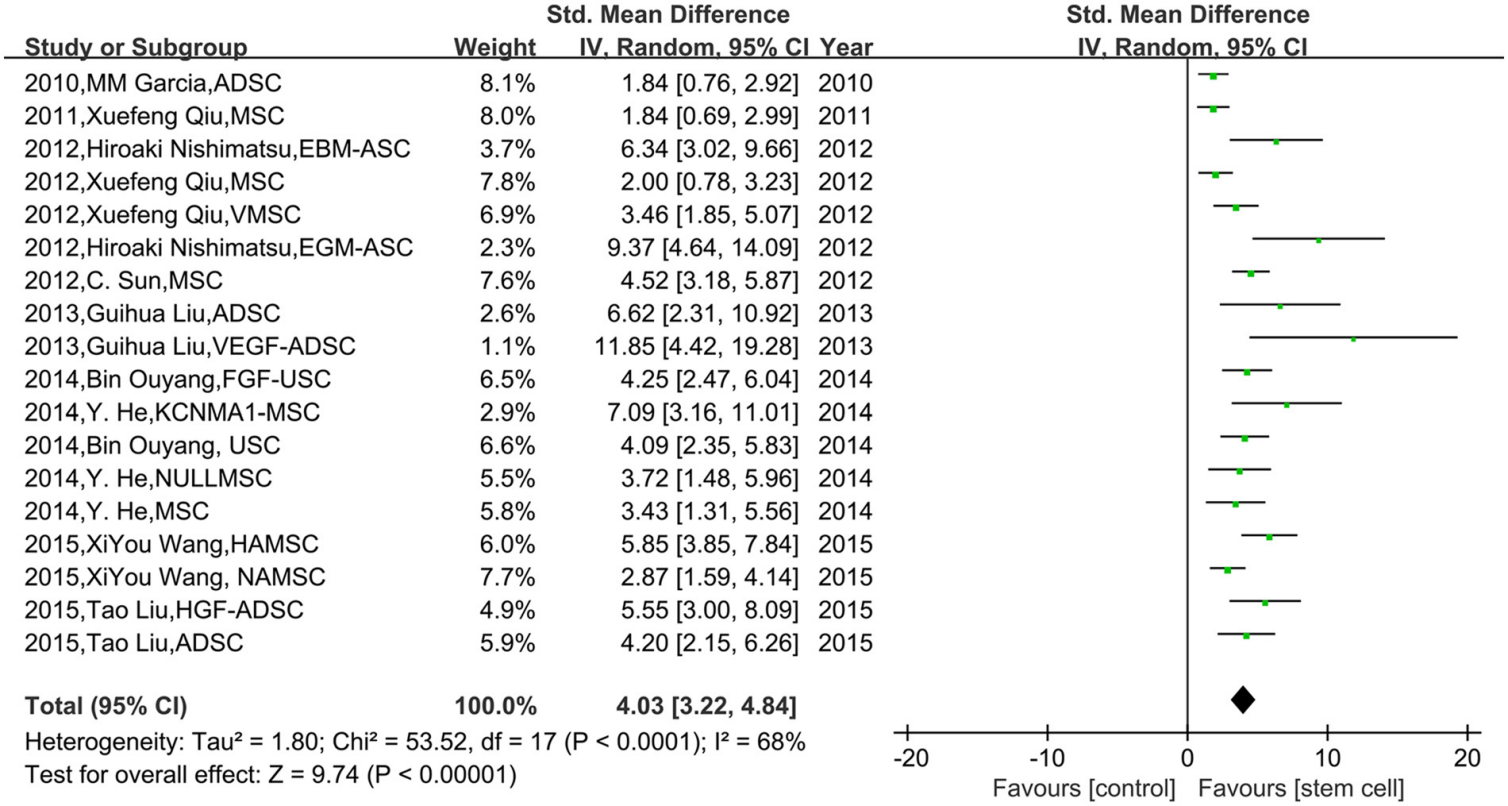
The effect of stem cells on ICP/MAP in diabetic rats. The random effects model forest plot graph shows that stem cell therapy could significantly improve the ICP/MAP compared with the control group.

A pooled analysis of all the included studies showed that stem cell therapy could significantly improve the erectile function compared with the control group (SMD 4.03, 95% CI = 3.22 to 4.84, *P* < 0.001, I^2^ = 68%; Fig 2).

**Fig 3:**
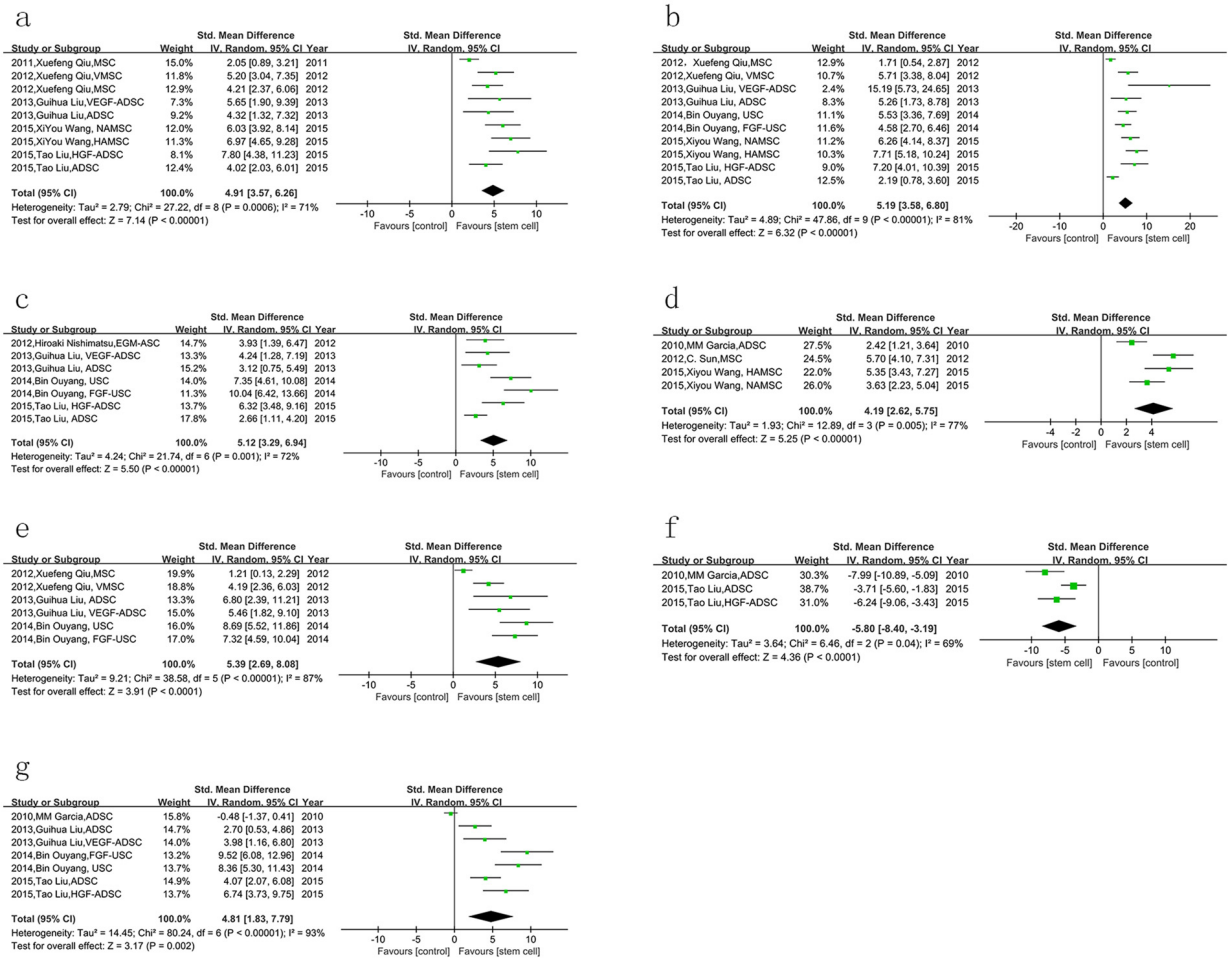
The effect of stem cells on changes in the structure and molecule of the cavernous body in diabetic rats. a. the smooth muscle content was increased by stem cell therapy; b. endothelium content was also increased; c. stem cell therapy could enhance the expression of eNOS; d. more expression of nNOS was detected in stem cell therapy group; e. stem cells lead to more secretion of VEGF; f. the apoptotic cells were reduced by stem cell therapy; g. stem cell therapy increased the ratio of smooth muscle to collagen in the cavernosum.

In order to clarify the underlying mechanism of stem cell therapy, we also analyzed the changes in the structure of the cavernous body between two groups. Both the smooth muscle (labeled with anti-α-SMA antibody) and endothelium (labeled with anti-CD31 antibody) content in the stem cell group were much more than those in control group (*P* < 0.001, Fig 3a and 3b). Moreover, the expression of eNOS and nNOS was also increased by stem cells (*P* < 0.001, Fig 3c and 3d). Vascular endothelial growth factor (VEGF) is a strong angiogenic reagent. Our results showed that stem cell therapy could repair the secretion of VEGF in the corpus cavernosum of rats with diabetic ED (*P* < 0.001, Fig 3e). Some studies assessed the cell apoptosis in the cavernous body with transferase dUTP nick end labeling (TUNEL) staining. The result of meta-analysis indicated that apoptotic cells were reduced significantly by stem cell treatment (*P* < 0.001, Fig 3f). Besides, we also performed meta-analysis of the results of Masson staining from four studies, which was used to detect the smooth muscle and collagen deposition in corpus cavernosum. SMC-to-collagen ratio was increased in the stem cell therapy group compared with control group (*P* = 0.002, Fig 3g). However, one of the four studies in this group showed that there was no significant difference in the Masson staining between two groups.

**Fig 4:**
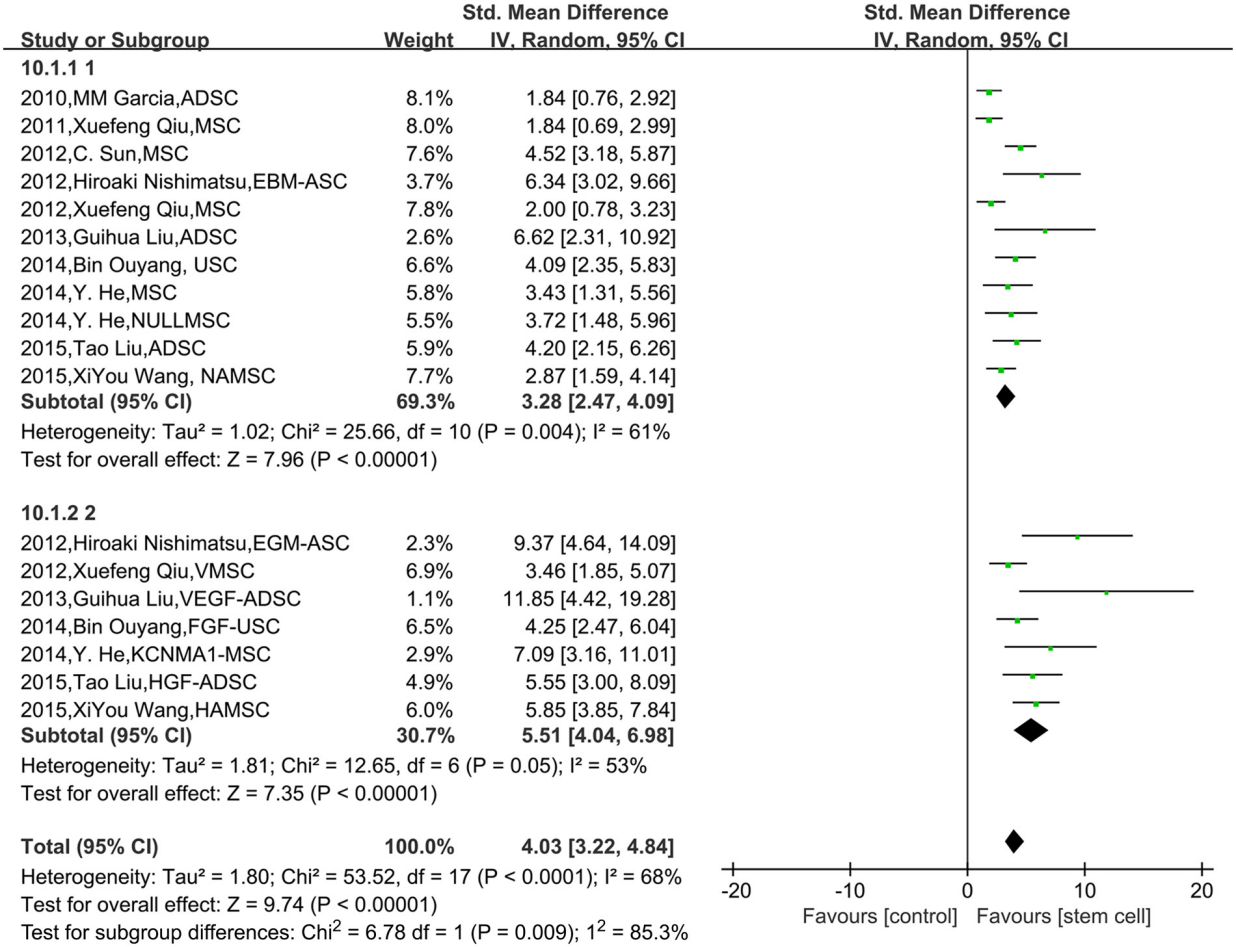
The comparison of the effects on ICP/MAP between modified stem cells and unmodified stem cells. The results of subgroup analysis showed that the modified stem cells were more effective than the unmodified ones in improving ICP/MAP, P = 0.009.

**Fig 5:**
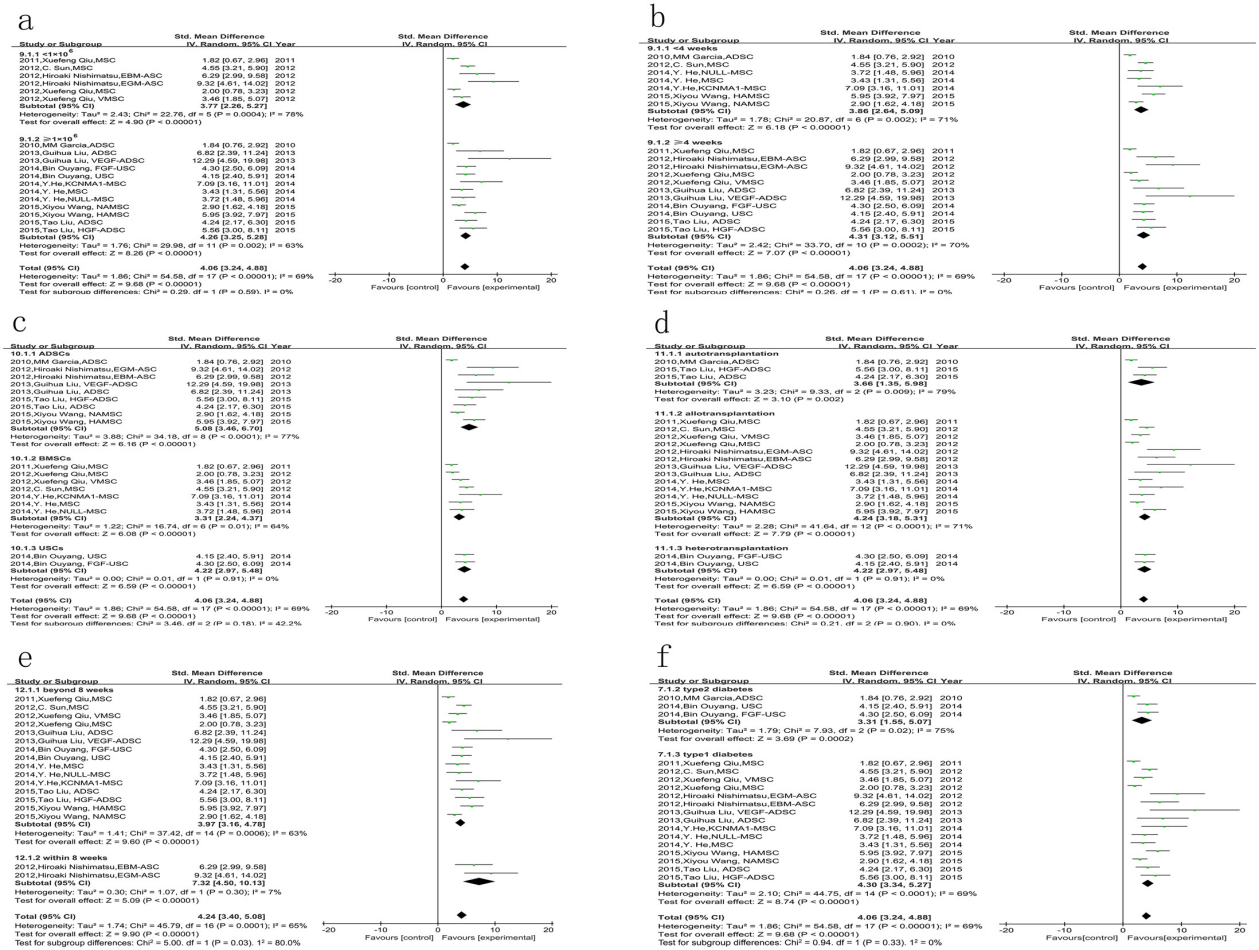
The comparison of the effects on ICP/MAP among different subgroups. There was no significant difference between the following subgroups: a. number of injected cells <1×10^6^ vs. ≥1×10^6^, P = 0.59; b. follow-up time after stem cell injection <4 weeks vs. ≥4 weeks, P = 0.61; c. stem cell types of ADSCs vs. BMSCs vs. USCs, P = 0.18; d. autotransplantation vs. allotransplantation vs. heterotransplantation, P = 0. 90; f. type1 diabetes vs. type 2 diabetes, P = 0. 33;. e. but the ICP/MAP was higher in the group with the injection of stem cells within 8 weeks than the group beyond 8 weeks, P<0.05.

The results of subgroup analysis showed that the modified stem cells were more effective than the unmodified ones in improving erectile function (*P* = 0.009, Fig 4). But there was no significant difference between the following subgroups: number of injected cells <1×10^6^ vs. ≥1×10^6^ (*P* = 0.59, Fig 5a); follow-up time after stem cell injection <4 weeks vs. ≥4 weeks (*P* = 0.61, Fig 5b); stem cell types of ADSCs vs. BMSCs vs. USCs (*P* = 0.18, Fig 5c); autotransplantation vs. allotransplantation vs. heterotransplantation (*P* = 0. 90, Fig 5d); and type1 diabetes vs. type 2 diabetes (*P* = 0. 33, Fig 5f).

The time point of stem-cell injection after the diabetic model establishment may have some influences on the efficacy. The subgroup analysis indicated that the ICP/MAP was higher in the group with the injection of stem cells within 8 weeks than the group beyond 8 weeks (*P*<0.05, Fig 5e). But there was only one study in the former group while 8 studies in the latter one, thus this result needs further investigation.

**Fig 6:**
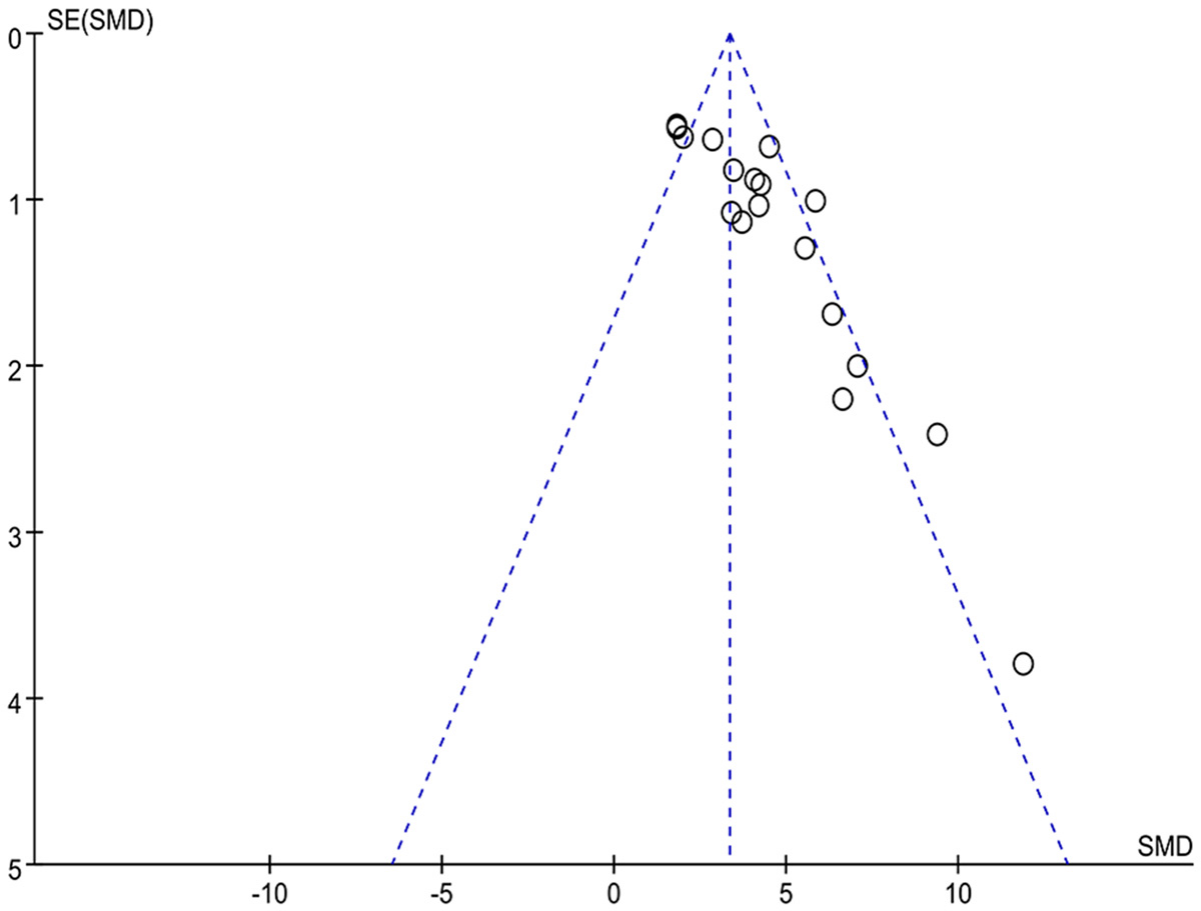
Funnel plot for the publication bias test of ICP/MAP.

The funnel plot (Fig 6) showed an apparent asymmetry, which suggested the existence of a potential publication bias.

## Discussion

The current meta-analysis examined 10 preclinical studies of stem cell therapy in the treatment of erectile dysfunction in diabetic rats. Overall, treatment with stem cells could improve the diabetic ED, which was evaluated by ICP/MAP.

Stem cells are potential of differentiating into various kinds of cells and tissues. In addition, they are capable of secreting different types of cytokines, such as VEGF and brain-derived neurotrophic factor (BDNF), which can protect cells from apoptosis and promote cell survival [18]. It was reported [8] that BMSCs could differentiate into cells expressing endothelium and smooth muscle markers after intracavernous transplantation, meanwhile several studies in our meta-analysis showed that the concentration of intracavernous VEGF was increased after stem cell therapy. This indicated that both the above mentioned effects of stem cells may combine to improve erectile dysfunction. But the paracrine action of stem cells might play the pivotal role, because all the included studies showed that no or only a few stem cells were detected in the cavernosum at the time of measuring ICP. The pathogenesis of diabetic erectile dysfunction involves the impairments in the endothelium of blood vessels, smooth muscle and nerve function [19]. Our meta-analysis showed that the smooth muscle and endothelial cells content, which were labeled with anti-α-SMA antibody and anti-CD31 antibody respectively, were both increased by stem cell therapy. In addition, Study of C. Sun et al. [14] indicated that nerve fibres within penile dorsal nerve were also promoted by intracavernous injection of BMSCs. These demonstrated that stem cell therapy could protect the fundamental structure of cavernosum in diabetic conditions.

It is well known that nitric oxide (NO) plays a pivotal role in the process of normal erection, which is synthesized mainly by nNOS and eNOS [20]. Angulo et al. [21] reported that (NO)/cyclic guanosin monophosphate (cGMP) pathway was impaired in patients with ED, especially diabetic ED. Our results showed that the expression of nNOS and eNOS in cavernous body could be improved by the stem cells in diabetic rats. This indicated that stem cell therapy could protect neuronal and endothelial function of the penis in diabetic rats. However, whether the NO/cGMP signaling was also increased demands more research.

Besides, two included studies examined the apoptosis of cells in the penis. Although our meta-analysis showed that stem cell therapy could reduce the number of apoptotic cells, the result remains to be further investigated with more studies.

VEGF is an angiogenic factor, which can stimulate the growth and proliferation of not only endothelial cells but also various other cells in corpus cavernosum [22]. Our meta-analysis demonstrated that stem cell therapy could increase the VEGF concentration in the cavernous body. This may partly explain the mechanism of stem cell therapy.

Reduced smooth muscle content with an excess deposition of collagen is another important etiology for diabetic erectile dysfunction [23]. Stem cells were shown to increase the ratio of SMC to collagen in our meta-analysis. But there was one study with a different result, which revealed no significant difference in smooth-muscle/collagen ratio between stem cell therapy group and control group. This controversy remains to be studied in the future.

From these data we could speculate that the potential mechanisms of stem cell therapy were as follows: On the one hand, injected stem cells differentiate into different kinds of cells in the cavernosum, such as endothelium, smooth muscle and nerve fibers. On the other hand, these stem cells secreted various growth factors like VEGF and BDNF, which were able to promote the survival of intracavernous component cells in pathological conditions. The latter may play a more important role [24]. As a result, the expression of nNOS and eNOS was increased, activating the NO/cGMP pathway, thereby improving the erectile function.

There were many differences among the included studies, which might lead to different therapeutic effects. It is necessary to indentify the best therapy strategy. The results of our subgroup analysis indicated that the modified stem cells were more effective in increasing ICP/MAP than the unmodified ones. The main modification method is transfection with viruses expressing growth factor genes, such as VEGF, FGF2 and HGF, which are able to promote the survival of various cells in diabetic condition, including the stem cells themselves. KCNMA1 gene modification can also enhance the efficacy of stem cells [15]. Its expression can cause intracellular K^+^ outflow, membrane hyperpolarization and then decrease the excitability of cell [25]. In this way, it could reduce the contraction of vascular smooth muscle cells, therefore improving the erectile function. Hence we have reasons to believe that the stem cell therapy combined with gene therapy could be a more effective treatment for erectile dysfunction. In addition, culture of stem cells in endothelial growth medium containing various kinds of growth factors has the similar effects. Another modification approach is hypoxic preconditioning, which was demonstrated to increase the expression of defense genes in stem cells [26]. In fact, there are many methods of stem cell modification and appropriate modification could enhance the protective effects of stem cells. However, which is optimal among these different kinds of modification still needs more research. Because the number of studies included in our meta-analysis was limited and each study used a distinct modification method, it was not appropriate to perform a subgroup analysis.

It is worth noting that three of the included studies used only unmodified stem cells while seven used both unmodified and modified stem cells. In these seven studies, the unmodified stem cells could be used as control for modified cells to demonstrate that the modification was effective. In order to investigate whether this could bring a bias of the target, we also performed a subgroup analysis to compare the efficacy of unmodified stem-cell therapies between these two different kinds of studies. The result showed that there was no significant difference in ICP/MAP between the rats receiving unmodified cells as the experimental arm and those receiving unmodified cells as control for modified cells (data not shown).

Early intervention may also be a way to increase the efficacy of stem cell therapy. The subgroup analysis showed that the administration of stem cells in 6 weeks after diabetes formation could better improve ICP/MAP compared with that beyond 8 weeks. However, there was only one study performed early intervention. Therefore, the result was not reliable and must be further investigated. The study of MM. Garcia was not included in this subgroup analysis, because that its injection time was at the age of 23 weeks of ZDF rats. This measurement method was different from other studies and would produce bias if included.

Other factors, such as the type of stem cells, number of injected cells, follow-up time after stem cell therapy, stem cell sources and diabetes types may not influence the effect of stem cell therapy on erectile function significantly. These were similar with results from the study of Haitao Shan, et al. [17].

Although stem cell therapy is an efficient method to treat diabetic erectile function, it could not completely recover the impaired erectile function in diabetic rats. Most studies showed that the ICP/MAP in unmodified stem-cell therapy group was about 60% of that in normal control group, while in the modified stem-cell therapy group it was approximately 80%. Among them, studies of Hiroaki Nishimatsu and Bin Ouyang showed the best therapeutic effect, which used EGM-modified adipose-derived stem cells and FGF2-modified urine-derived stem cells respectively. But which kind of stem cell therapy is optimal still demands further investigation.

Another unresolved question is that how long the efficacy of stem-cell therapy could last. We could not give the answer at the moment, because the longest follow-up time in these included studies was 1 month. But it was reported [27] that human umbilical cord blood stem cells combined with PDE5 inhibitors could recover the erectile function in patients with type 2 diabetics for more than 6 months. This indicated that stem cell therapy was effective in humans and that its efficacy was durable, which bring us new hope for further research on stem cell therapy.

## Limitations

10 studies with a median quality score of 6 of 9 were included in our meta-analysis. Although these studies were of relatively high quality, there were many differences among studies, such as modification of stem cells, cell numbers and follow-up time, which lead to statistically significant heterogeneity. In order to minimize the risk of erroneous estimates, we used random effects analysis to perform the meta-analysis. Besides, only 10 articles met the inclusion criteria and the sample sizes of these were not large enough to give convincing evidence.

Another limitation is that the meta-analysis of experimental studies is generally susceptible to publication bias due to unpublished negative studies. All the studies included in our meta-analysis showed the protective effects of stem cell therapy in erectile function. We have tried our best to collect all related studies, but could not avoid the publication bias. This may exaggerate the therapeutic effect of stem cell therapy. So we should elucidate the results of this meta-analysis cautiously and need further research to confirm them. At last, some of the studies did not present the outcomes with detailed data and we were not able to get responses from the authors. We could only obtain these data by digitizing the column graphs, which may influence the accuracy of data. However, two authors of our study extracted these data separately in order to increase the reliability.

## Conclusions

Stem cell therapy has been a novel therapy for many diseases recently. As far as we know, this meta-analysis is the first one to evaluate the efficacy of stem cell therapy on diabetic ED in rats. Our results confirm that stem cell therapy can apparently improve the erectile function of diabetic rats. The possible mechanism of this effect may include the increase of the content of smooth muscle and endothelium, the up-regulation of nNOS and eNOS expression, the enhanced secretion of VEGF, as well as the decrease of fibrosis and apoptosis. Moreover, some specific modification, especially the gene modification with growth factors, could improve the efficacy of stem cell therapy, but the stem cell numbers, follow-up time, stem cell types, stem cell sources and diabetes types may have no influence. These findings indicate that stem cell therapy has the potential to be an effective therapeutic strategy. In addition, our research may be helpful for the design of future experimental studies.

## Supporting Information

S1 File PRISMA Checklist. (PDF)

S2 File Search Strategy. (PDF)

S3 File Included and Excluded Studies. (PDF)
