# Supplementary material for: Stem Cell Therapy for Diabetic Erectile Dysfunction in Rats: A Meta-Analysis
Source: PLoS One. 2016 Apr 25;11(4):e0154341. doi: 10.1371/journal.pone.0154341 (PMC4844188; doi:10.1371/journal.pone.0154341)
Supplement: S2 File — (PDF) [file pone.0154341.s002.pdf]

We searched the pre-clinical studies analyzing the efficacy of stem cell therapy for diabetic ED published before September 30, 2015 in PubMed, Web of Science and EBSCO. The following search strategy was used: (Erectile dysfunction) and (stem cell) and (diabetes) and (rat)
