# Supplementary material for: Stem Cell Therapy for Diabetic Erectile Dysfunction in Rats: A Meta-Analysis
Source: PLoS One. 2016 Apr 25;11(4):e0154341. doi: 10.1371/journal.pone.0154341 (PMC4844188; doi:10.1371/journal.pone.0154341)
Supplement: S3 File — (PDF) [file pone.0154341.s003.pdf]

Included Studies:

Treatment of erectile dysfunction in the obese type 2 diabetic ZDF rat with adipose tissue-derived stem cells

Intracavernous Transplantation of Bone Marrow-Derived Mesenchymal Stem Cells Restores Erectile Function of Streptozocin-Induced Diabetic Rats

Neurotrophic effect of bone marrow mesenchymal stem cells for erectile dysfunction in diabetic rats

Adrenomedullin Mediates Adipose Tissue-Derived Stem Cell-induced Restoration of Erectile Function in Diabetic Rats

Combined Strategy of Mesenchymal Stem Cell Injection With Vascular Endothelial Growth Factor Gene Therapy for the Treatment of Diabetes-Associated Erectile Dysfunction

Correction of Diabetic Erectile Dysfunction with Adipose Derived Stem Cells Modified with the Vascular Endothelial Growth Factor Gene in a Rodent Diabetic Model

Human Urine-Derived Stem Cells Alone or Genetically Modified with FGF2 Improve Type 2 Diabetic Erectile Dysfunction in a Rat Model

Transplantation KCNMA1 modified bone marrow mesenchymal stem cell therapy for diabetes mellitusinduced erectile dysfunction

Hepatocyte growth factor-modified adipose tissue-derived stem cells improve erectile function in streptozotocin-induced diabetic rats

Hypoxia Precondition Promotes Adipose Derived Mesenchymal Stem Cells Based Repair of Diabetic Erectile Dysfunction via Augmenting Angiogenesis and Neuroprotection

Excluded Studies:

10 were reviews:

Current Literature Review

Stem Cell Therapy for Erectile Dysfunction A Critical Review

Stem-cell therapy for erectile dysfunction

Vascular regenerative therapies for the treatment of erectile dysfunction: current approaches

Stem cell therapy for voiding and erectile dysfunction

Multipotent Stromal Cell Therapy for Cavernous Nerve Injury-Induced Erectile Dysfunction

RhoA/Rho-kinase and vascular diseases: what is the link?

Gene and stem cell therapy for erectile dysfunction

Emerging Molecular Targets for Treatment of Erectile Dysfunction: Vascular and Regenerative Therapies on the Horizon

Mechanisms of penile fibrosis

4 did not use stem cell therapy:

Neuromedin B Restores Erectile Function by Protecting the Cavernous Body and the Nitrergic Nerves from Injury in a Diabetic Rat Model

Effects of Low-Energy Shockwave Therapy on the Erectile Function and Tissue of a Diabetic Rat Model
Mobilisation of endothelial progenitor cells one of the possible mechanisms involved in the chronic administration of melatonin preventing erectile dysfunction in diabetic rats
Effects of Icariin on Improving Erectile Function in Streptozotocin-Induced Diabetic Rats

2 were not about diabetic ED:

Adipose Tissue-Derived Stem Cells Ameliorate Diabetic Bladder Dysfunction in a Type II DiabeticRat Model
Effects of sildenafil and/or muscle derived stem cells on myocardial infarction

3 used mice as objects of study:

Xenogenic Transplantation of Human Breast Adipose-Derived Stromal Vascular Fraction Enhances Recovery of Erectile Function in Diabetic Mice
Intracavernous Delivery of Freshly Isolated Stromal Vascular Fraction
Rescues Erectile Function by Enhancing Endothelial Regeneration in the Streptozotocin-Induced Diabetic Mouse
Angiotensin-(1-7) Reverses Angiogenic Dysfunction In Corpus Cavernosum By Acting On The Microvasculature And Bone Marrow-Derived Cells In Diabetes

1 was conference paper:

Skeletal Muscle Derived Stem Cells (MDSC) Ameliorate Erectile Dysfunction In A Rat Model of Type 2 Diabetes, But Their Repair Ability Is Severely Impaired When Isolated From Long-Term Diabetic Milieu

2 without ICP/MAP assessment:

Superoxide Dismutase – A Target for Gene Therapeutic Approach to Reduce Oxidative Stress in Erectile Dysfunction
Transplantation of endothelial progenitor cells transfected with VEGF165 to restore erectile function in diabetic rats

1 did not mention the procedure of the experiment:

Flk-1+Sca-1- mesenchymal stem cells: functional characteristics in vitro and regenerative capacity in vivo
